# Genome Assembly and Microsatellite Marker Development Using Illumina and PacBio Sequencing in the *Carex pumila* (Cyperaceae) from Korea

**DOI:** 10.3390/genes14112063

**Published:** 2023-11-10

**Authors:** Kang-Rae Kim, Jeong-Nam Yu, Jeong Min Hong, Sun-Yu Kim, So Young Park

**Affiliations:** Animal & Plant Research Department, Nakdonggang National Institute of Biological Resources, Sangju 37242, Republic of Korea; kimkangrae9586@gmail.com (K.-R.K.); susia000@nnibr.re.kr (J.-N.Y.); hjmjulia@nnibr.re.kr (J.M.H.); ksuny007@nnibr.re.kr (S.-Y.K.)

**Keywords:** genomics, genome assembly, medicinal plants, next-generation sequencing, halophyte plants

## Abstract

This study is the first to report the characterization of *Carex pumila* genomic information. Assembly of the genome generated a draft of *C. pumila* based on PacBio Sequel II and Illumina paired-end sequencing, which was assembled from 2941 contigs with an estimated genome size of 0.346 Gb. The estimate of repeats in the genome was 31.0%, and heterozygosity ranged from 0.426 to 0.441%. The integrity evaluation of the assembly revealed 1481 complete benchmarked universal single-copy orthologs (BUSCO) (91.76%), indicating the high quality of the draft assembly. A total of 23,402 protein-coding genes were successfully predicted and annotated in the protein database. UpsetR plots showed that 7481 orthogroups were shared by all species. The phylogenetic tree showed that *C. pumila* is a close but distant relative of *Ananas comosus*. *C. pumila* had greater contraction (3154) than expansion (392). Among the extended gene families, aquaporins have been found to be enriched. Primers for microsatellite markers determined 30 polymorphic markers out of 100. The average number of alleles amplified by these 30 polymorphic markers was 4 to 12, with an average polymorphism information content (PIC) value of 0.660. In conclusion, our study provides a useful resource for comparative genomics, phylogeny, and future population studies of *C. pumila*.

## 1. Introduction

The Cyperaceae family is a cosmopolitan plant family with medicinal properties, with about 5000 species distributed worldwide [1,2]. The Cyperaceae family consists of medicinal plants such as *Cyperus rotundus*, *Cyperus papyrus*, and *Carex baccans.* The plant has been reported over the past few decades to exhibit excellent chemical versatility and traditional medicinal and anticancer, anti-inflammatory, antibacterial, and anthelmintic properties [1,2,3,4].

*C. pumila* is widely distributed in Australia (e.g., Lord Howe Island), New Zealand, Chile, China, Japan, and Republic of Korea and inhabits salty coastal dunes [5]. Salt plants synthesize various bioactive molecules in response to survival mechanisms to survive in extreme environments [6]. In recent studies, *C. pumila* has been reported to have potential effects in inhibiting IgE-mediated allergic reactions, and invasion and metastasis of cancer cells [5,7]. To use it as an effective medicinal plant, it is important to cultivate *C. pumila* into cultivars. For faster growth and more medicinal enrichment, cultivar development is required, and genomic research can help develop new cultivars [8]. In addition, genomic information can be used to facilitate drug discovery in medicinal plants [9]. So far, four genome assemblies of the Cyperaceae family have been reported: *Kobresia littledalei*, *Carex parvula*, *Carex kokanica*, and *Kobresia myosuroides* [10,11,12,13]. Currently, genomic information is continuously increasing, but it has been less compared to the number of species of Cyperaceae. No research has been reported on the genome information of *C. pumila*.

Next-generation sequencing technology (NGS) is less expensive and more accurate than existing technologies. However, in the case of short-read sequencing, accuracy is high, but it has a challenge resolving repetitive regions. As the long-read technology develops simultaneously with the development of current technology, the problem of the repeat area is being solved [14]. By complementing the second generation short-read sequencing and third generation long-read sequencing technologies, precise genome information can be obtained with higher accuracy [15].

As a marker used for breeding, microsatellite, a related marker, is used. Microsatellites are often used for breeding selection due to their ease and high reproducibility as an associated marker, which is derived from their diversity of alleles and rich distribution within the genome [16]. In the Cyperaceae family, microsatellite markers have been developed in species such as *Carex macrocephala*, *Carex scoparia*, *Carex curvula*, *Carex eburnea*, *Carex kobomugi*, *Schoenoplectus americanus*, *Lepidosperma gibsonii*, *Baumea juncea*, and *Cyperus fuscus* [17,18,19,20,21,22,23,24,25]. However, *C. pumila* microsatellite markers have not been developed to date.

In this study, we generated a draft genome through sequencing and assembly of *C. pumila*. We annotated genes, predicted protein-coding genes, determined phylogenetic positions using orthogroups, and developed microsatellite markers. This study provides an important resource for future population genetic studies and molecular breeding using the developed markers.

## 2. Materials and Methods

### 2.1. Sample Preparation

**Figure 1:**
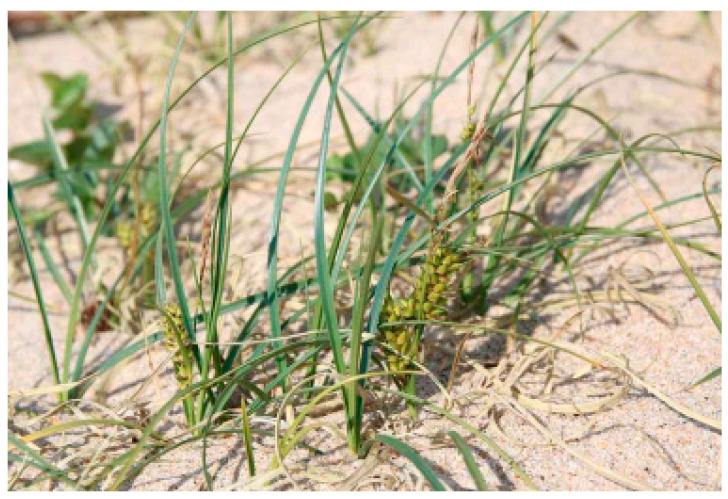
One photograph of specimens of *C. pumila* used in the study.

**Figure 2:**
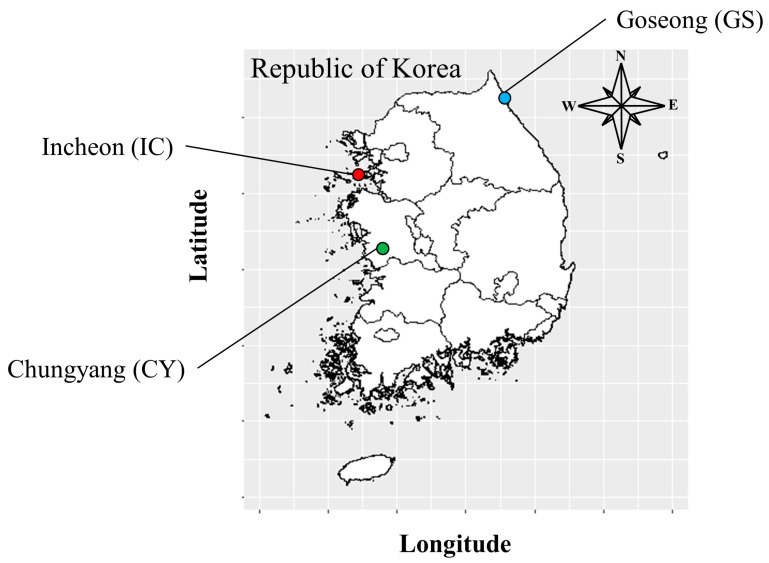
Location of sites for *C. pumila* sample collection. The samples were collected from Goseoung (GS), Incheon (IC), and Chungyang (CY).

Samples were collected from three wild populations located in the Republic of Korea during 2022 (Figure 1 and Figure 2) (Goseoung (GS; 38°22′01.00″ N, 128°30′32.2″ E), Incheon (IC; 37°14′50.2″ N, 126°31′27.7″ E), and Chungyang (CY: 36°19′14.27″ N, 126°56′16.16″ E)). Leaf samples for NGS analysis were collected from one Goseong population. The collected samples were dried using silica gel and stored in a deep freezer (−80 °C). Eight individuals from three populations were sampled for microsatellite marker polymorphism analysis. Genomic DNA was extracted from the stored samples using the DNEasy Plant Mini Kit (QIAGEN, Hilden, Germany) according to the manufacturer’s protocol. The quality of the extracted genomic DNA was checked using a 1% agarose gel and a Nanodrop spectrophotometer. Total RNA was extracted from leaf tissue using the RNeasy Plant Mini Kit (QIAGEN) according to the manufacturer’s protocol (Macrogen, Inc., Seoul, Republic of Korea).

### 2.2. Genome Sequencing and De Novo Assembly

The whole genome was sequenced using a 150 bp paired-end library produced by Macrogen (Macrogen Inc., Seoul, Republic of Korea) and using the Illumina HiSeq 2500 (Illumina, San Diego, CA, USA) platform. In addition, to solve the serial repeat problem in plants, we used single-molecule real-time sequencing from Pacific BioSciences (PacBio) following a protocol from Macrogen (Macrogen Inc., Seoul, Republic of Korea). DNA sequencing libraries were prepared and contiguous long reads (CLRs) obtained using the PacBio RS II DNA sequencer platform. Initial assembly was run with default parameter values using HGAP ver. 3.0 [26] and Wtdbg2 ver. 2.3 [27]. Long sequences were assembled by merging similar segments into vertices and connecting reads based on segment adjacency. The Illumina short-read run with default parameters using the Trimmomatic ver. 0.33 [28]. Clean reads were obtained after removing adapter sequences. To improve the accuracy of initial contig assembly, BWA-MEM [29] and Pilon version 1.23 [30] were used with default parameters. The draft genomes generated in two assemblers for the integrity of the assembly with Embryophyta odb version 10 using Benchmarking Universal Single-Copy Orthologs ver. 5.2.2 [31]. Of the two genomes, we used the genome from HGAP and with the best results for BUSCO and N50 values. The assembled genome has been deposited in NCBI GenBank whole-genome shotgun database under accession number JARJHM000000000.

RNA Iso-seq libraries were prepared for genome annotation and sequenced using the PacBio sequel II platform according to the manufacturer’s protocol (Macrogen Inc., Seoul, Republic of Korea). Iso-seq results yielded a total of 18,257,235 subreads (Table 1).

Default parameter values from Jellyfish (ver. 2.3.0) [32] and GenomeScope (ver. 1) [33] were used to estimate genome size, heterozygosity, and repeatability.

### 2.3. Genome Annotations and Gene Ontology Analysis

Putative protein-coding genes in the genome sequence were predicted using the MAKER pipeline [34], using predicted Iso-Seq data of *C. pumila* and peptide sequences from the genomes of *Oryza brachyantha*, *Oryza sativa*, *Zea mays*, and *Panicum miliaceum*. Two training sets, AUGUSTUS and SNAP, were also used for prediction, and sequences with an annotation edit distance score less than 0.5 were selected as high-confidence genes. Further annotation of consensus sequences against the InterPro (ver. 69.0, https://www.ebi.ac.uk/interpro/, accessed on 1 January 2022), Pfam (ver. 31.0, http://pfam.xfam.org/, accessed on 1 January 2022), and EggNOG (ver. 4.5, http://eggnog.embl.de, accessed on 1 January 2022) databases was performed using BLAST (ver. 2.7.1+, BLAST e-value: 1.0 × 10^−5^). To search for functional classification of annotations, gene ontology (GO) and clusters of orthologous group (COG) annotations were performed using OmicsBox (https://www.biobam.com/omicsbox, accessed on 1 January 2023) software with default parameters and the results were exported to WEGO software ver. 2.0. These results were further analyzed using Web Gene Ontology Annotation Plot (http://wego.genomics.org.cn/cgi-bin/wego/index.pl, accessed on 1 January 2022). KEGG (Kyoto Encyclopedia of Genes and Genomes) annotations were used for KEGG pathway (https://www.genome.jp/kegg/, accessed on 1 January 2022) mapping using KAAS [35].

### 2.4. Phylogenetic Tree Reconstruction

A total of 25 protein sequences derived from genomes were compared, including *C. pumila*, to other plant species. Protein sequences were downloaded from NCBI and used for analysis (Table S2). Protein sequences were obtained from OrthoFinder ver. 2.5.4 [36] to organize the groups of orthologous genes. A matrix of the number of genes in orthogroups were calculated for each plant species and used with UpSetR [37]. After selecting the significant groups with *p* < 0.01 from the phylogenetic tree using CAFE5 ver. 5.0.0 [38], orthogroup expansion and contraction were measured using CafePlotter (https://github.com/moshi4/CafePlotter, accessed on 1 January 2022), and genes were reconstructed.

### 2.5. Analysis of Microsatellite Markers and Genotyping

Microsatellite screening was performed using the MIcroSAtellite (MISA) tool (https://webblast.ipk-gatersleben.de/misa/, accessed on 1 January 2022). Parameters for di-, tri-, tetra-, penta-, and hexa-nucleotides were set at 10, 4, 4, 4, and over 4 repeats, respectively. For each microsatellite locus, primers were designed according to the following five parameters using Primer3 software (https://github.com/primer3-org/primer3, accessed on 1 January 2022): amplicon length 100–400 bp, primer size 20–24 bp, GC content 40–60%, and melting temperature 57–59 °C. For the designed microsatellite primers, the presence of multiple binding regions in contigs was confirmed using SnapGene (GSL Biotech, Chicago, IL, USA, https://www.snapgene.com/, accessed on 1 January 2022).

Microsatellite loci amplification was performed using the Mastercycler^®^ Pro Gene Amplifier. The polymerase chain reaction was performed with a total volume of 20 µL using H-Star Taq DNA Polymerase (Biofact, Daejeon, Republic of Korea). PCR fluorescent labeling was performed according to the protocol described by Schuelke (2000) [39] using four fluorescent primers: microsatellite-specific forward primer (0.4 μM), reverse microsatellite synthesized with an M13 (TGTAAAACGACGGCCAGT) tail at the 5’ end, specific primer (0.8 μM), and fluorescent-labeled (6-FAM, VIC, NED, PET) M13 primer (0.4 μM). The PCR conditions were 94 °C for 5 min, 94 °C for 30 s, 56 °C for 45 s, 72 °C for 45 s, 94 °C for 30 s, 53 °C for 45 s, 12 cycles at 72 °C for 45 s, and 72 °C for 7 min. For microsatellite PCR amplification products, GeneScan™ 500 ROX Size Standard Ladder (Applied Biosystems, Foster City, CA, USA) was mixed with HiDi™ formamide, denatured at 95 °C for 2 min and incubated at 4 °C. Allelic sizes were determined using an ABI 3730xl DNA Analyzer (Applied Biosystems). Genotyping was determined using the GeneMarker^®^ 2.6.7 program (SoftGenetics, State College, PA, USA).

To evaluate the usefulness of the developed microsatellite loci, polymorphism information content (PIC), number of alleles, predicted heterozygotes (*H*_E_), and observed heterozygotes (*H*_O_) were analyzed using Cervus 3.0 software [40].

## 3. Results and Discussion

### 3.1. Genome Assembly of C. pumila

For whole-genome sequencing, 870,146,384 reads were generated using Illumina short-read sequencing, and the total read length was 131,392,103,984 bp. After removing adapter sequences and low-quality reads using Trimmomatic [28], the total number of clean reads was 350,433,302, and the total length of reads was 52,800,516,376 bp (Table 1). The Q20 and Q30 quality were 99.47% and 97.17%, respectively (Table 1). PacBio sequencing identified 33,136,805 subreads, with a total length of 127,339,898,260 bp and an average subread length of 3842 bp (Table 1). Genomes assembled from Wtdbg2 were not suitable because they had low numbers of contigs in contrast to low N50 values (N50: 160,342). The genome assembly using HGAP yielded 2941 contigs with a total length of 346,579,715 bp (Table 2). The length of the contigs ranged from 1,780,279 to 1345 bp (average 117,844 bp). The N50 value was 351,713 bp, and the average GC content of the *C. pumila* genome was 33.4% (Table 2). *C. parvula* and *C. kokanicas* had GC contents of 35.41% and 34.68%, which compared similarly with *C. pumila* [12].

**Figure 3:**
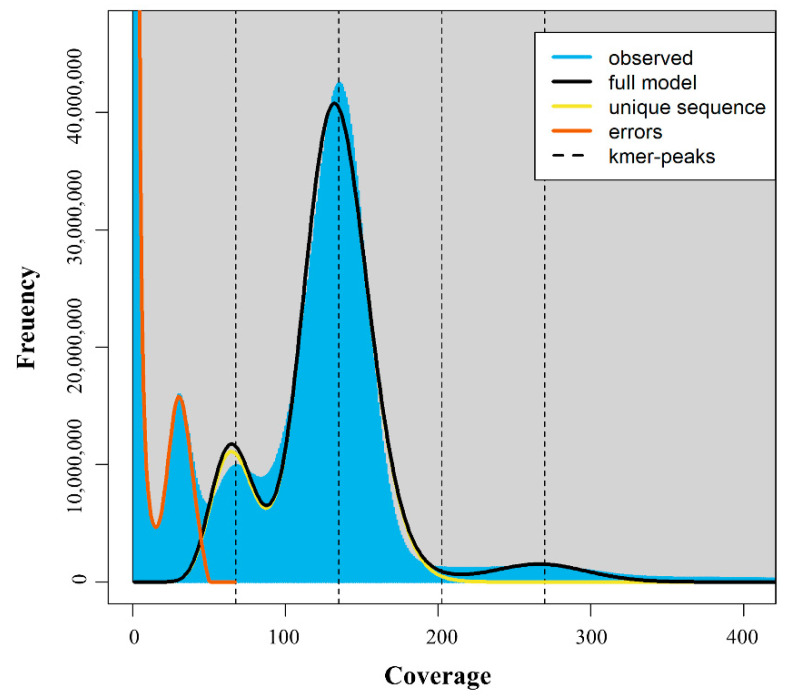
Estimation of genome size using k-mer peaks of *C. pumila*.

The k-mer distribution (Table S1) is shown in Figure 3 as a histogram constructed using Jellyfish software (ver. 2.3.0) [32]. The estimated haploid genome size is approximately 0.338 Gb, and the actual assembled genome size is 0.347 Gb. Although the estimated genome size of *C. pumila* appears small (C-value: 0.30; 0.58 Gb), the average genome size is similar to other *Carex* species in the Plant DNA C-value database (average 0.86 Gb; https://cvalues.science.kew.org, accessed on 1 January 2022). Although it falls short of the actual genome size, additional honing of long-read and short-read sequencing could improve genome size. Nonetheless, the draft genome from this study will help improve breeding and disease resistance.

Complete BUSCO analysis confirmed that HGAP (91.76%) had a more complete assembly genome than Wtdbg2 (86.12%). BUSCO (Embryophyta odb10, https://busco-archive.ezlab.org/frame_plants.html, accessed on 1 January 2022) analysis identified a total of 1614 BUSCO of HGAP (Table 3). Of these, 91.76% (1481) of the Embryophyta gene sequences were complete (Table 3), which comprised 1416 single-copy BUSCO (87.73%) and 65 duplicated-copy BUSCO (4.02%). The number of fragmented BUSCO was 31 (1.92%), and the number of missing BUSCO was 102 (6.32%). *C. parvula* and *C. kokanica*, which are related species of *C. pumila* (91.76%), showed high integrity compared to the genomes of related species at 85.9% and 88.2%, respectively, indicating a high quality of the genome [12].

### 3.2. Annotation of Candidate Genes and Protein Prediction

For protein-coding genes, MAKER was used, and 23,402 were predicted. The total length of these genes was 35,450,136 bp (Table 4). The number of protein-coding genes predicted in the *C. pumila* genome was lower than that predicted in the *C. parvula* (45,002) and *C. kokanica* (36,709) genomes [12]. This may be due to differences in the parameters and methods used for gene prediction, and rigorous prediction reduces the number of gene predictions. Genes annotation across various databases resulted in the identification of 22,464 (81.58%), 18,762 (95.99%), 19,190 (82.00%), 9741 (41.62%), and 8667 (37.04%) in the EggNOG, InterPro, Pfam, COG, and KEGG databases, respectively.

**Figure 4:**
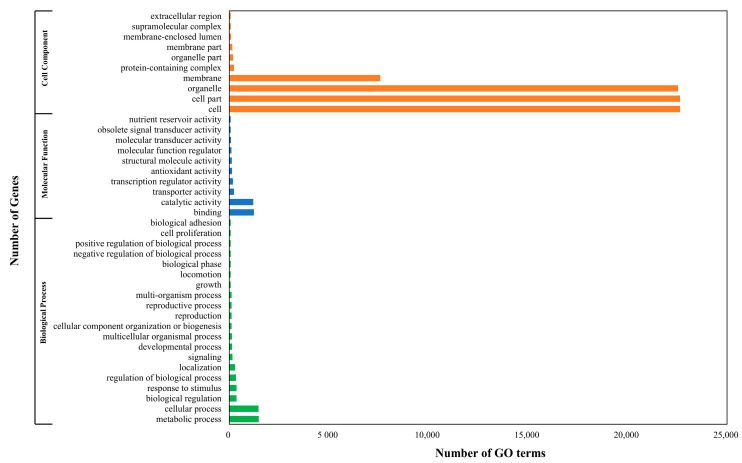
Gene ontology term annotation categories of level 3 for *C. pumila*: molecular functions, cellular components, and biological processes.

The GO annotation of the predicted coding sequence (CDS) identified all three major GO terms (Figure 4): cellular component (68.28%), molecular function (3.54%), and biological process (4.28%). Within the cellular component, most were assigned to “cell” (22,575, GO:0005623), “cell part” (22,574, GO:0044464), “organelle” (22,482, GO:0043226), and “membrane” (7519, GO:0016020); “binding” (1171, GO:0005488) and “catalytic activity” (1139, GO:0003824) within the molecular function; and “metabolic process” (1415, GO:0008152) and “cellular process” (1403, GO:0009987) within the biological process.

**Figure 5:**
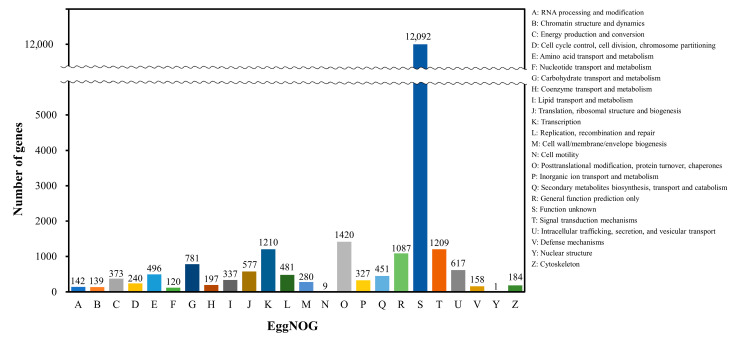
EggNOG functional classification information in *C. pumila*. Genes were assigned to 24 categories, excluding the “extracellular structure” category. The wave pattern in the diagram is omitted because the size of the numbers is too large.

In EggNOG, 22,464 genes were classified into 24 categories, excluding the extracellular structures category (Figure 5). Among these, the unknown function was the most common within 22,464 cases (95.99%, function unknown was 12,092; 52.74%). This was followed by posttranslational modification, protein turnover, chaperones at 1420 (6.19%), transcription at 1210 (5.28%), signal transduction mechanisms at 1209 (5.27%), and general function prediction only at 1087 (4.74%).

**Figure 6:**
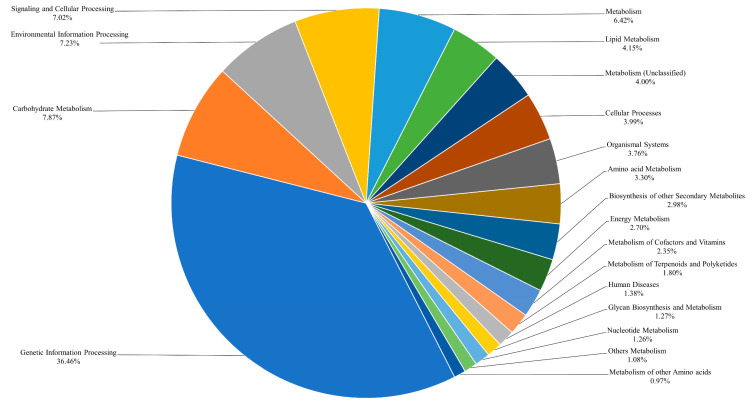
Percentage of KEGG ontology (KO) terms annotated in the *C. pumila* genetic dataset. Genes annotated in the Kyoto Encyclopedia of Genes and Genomes (KEGG) database are grouped into major functional categories based on the annotated pathways.

In KEGG orthologs, 8667 genes were annotated into 19 categories (Figure 6). Most of the genes annotated to “Genetic Information Processing” (36.46%), followed by “Carbohydrate Metabolism” (7.87%), “Environmental Information Processing” (7.23%), and “Signaling and Cellular Processing” (7.02%). Genes without annotations were classified as hypothetical proteins [40,41].

### 3.3. Phylogenetic Inference Orthologous Groups of C. pumila

**Figure 7:**
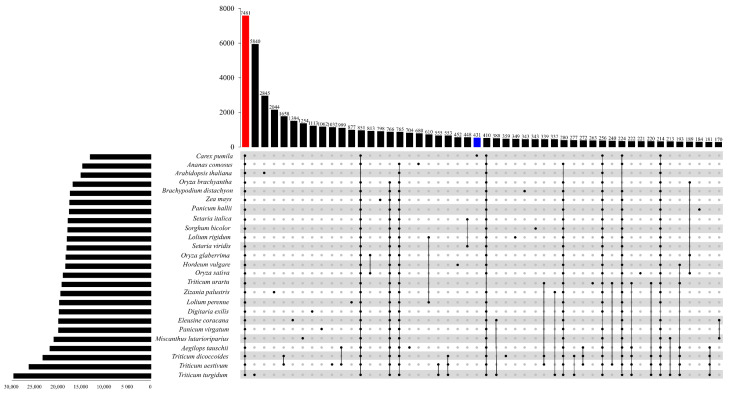
Orthogonal clusters of proteins from 25 reference genomes displayed as UpSet plots. Straight lines connected at each intersection point represent orthogonal groups that are shared. Blue bar: Orthogonal cluster for *Carex pumila*, red bar: Orthogonal cluster for all species.

We identified 60,498 orthogroups matching 1,418,513 genes using Orthofinder [36], including *C. pumila* and 25 species (Table S2). UpsetR plots showed that 7481 orthogroups were shared by all species (Figure 7). UpsetR plots showed 431 orthogroups specifically identified in *C. pumila*, and 765 orthogroups were shared by 25 species except *C. pumila*.

**Figure 8:**
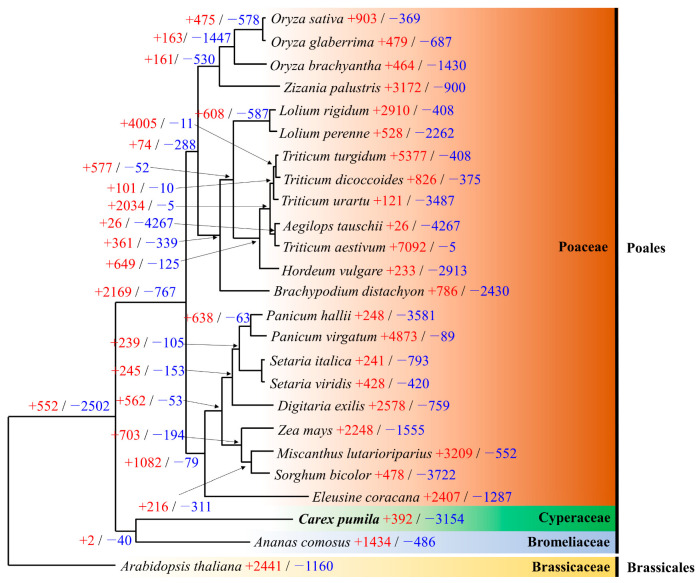
A phylogenetic tree of 25 species including Bromeliaceae, Brassicaceae, Poaceae, and Cyperaceae plants. Using CAFE5, gene family expansions (+) and contractions (−) were calculated at each ancestral node and divergence and species.

The phylogenetic tree showed that *C. pumila* was close to *A. comosus* but distantly related. This is because the two species differ at the family level. We analyzed the expansion and contraction of gene families based on the data of orthogroups generated by Orthofinder (Figure 8) and specifically based on the statistically significant values of orthologroups (*p* < 0.01). There were significant gene family expansions (2–4005) and contractions (5–4267) among the 25 plant genomes (*p* < 0.01). *C. pumila* showed more contraction (3154) than expansion (392).

The extended orthogroups are the cellular component: “membrane”, “organelle”, “intracellular organelle”; molecular function: “binding”, “catalytic activity”, “organic cyclic compound binding”; and biological process: “metabolic process” and “cellular process”, which were found to be significantly enriched (Table S3). Among them, aquaporin was also found to be abundant.

Aquaporin maintains efficient water transport in roots in a salt-stressed environment, resulting in high salinity tolerance [42]. It has been reported that the salt plant (*Puccinellia nuttalliana*) is also rich in aquaporin [42]. In addition, unlike other freshwater plants, *C. pumila* is presumed to be rich in aquaporins because it lives in the saline environments of sand dunes. This suggests that *C. pumila* has an expansion of genes related to salt tolerance, such as aquaporin, compared to other plants.

### 3.4. Development of Novel Microsatellite Markers for Population Study

We screened microsatellite regions in the assembled genome of *C. pumila*. The genome contained 62,565 microsatellite regions (Table 5). Di-nucleotides were the most common repeat motif (23,876; 38.16%), followed by tri- (23,857; 38.13%), tetra- (7617; 12.17%), penta- (3774; 6.03%), and hexa- (3441; 5.50%) nucleotides (Figure S1). AT repeats motif (62.44%) were most frequently distributed in di-nucleotides. AT/TA repeat motifs (62.44%) were most frequently distributed in di-nucleotides. It has also been observed in *Matthiola incana* [43], rice [44], *Fagopyrum tataricum* [45], and other species. This may be the reason for the higher number of slips in shorter repetitions [46]. In the tri-nucleotide, AAT/ATT repeat motifs accounted for 49.22%. Tri-nucleotides also showed a distribution similar to that of di-nucleotides, which may differ depending on the parameter value of microsatellite screening. Abundant repeats of AT in monocotyledonous plants are reported to be uncommon [47]. In *C. pumila*, the low GC contents are thought to be due to the low GC contents of the nucleic acids in the presence of the high frequency of A and T present in the genome.

Of the 62,565 identified microsatellites, 89.11% (55,696) were suitable for developing markers. We randomly selected 100 primer sets, of which only 50 (50.0%) primer sets were successfully amplified. Of these, 30 markers (60.0%) were polymorphic with a PIC value of 0.3 or higher. Finally, we selected 30 microsatellite markers amplifying various repeat motifs for the population study (Table 6). These 30 markers have been deposited to NCBI under the accession numbers OQ685862-OQ685891 (Table 6).

The number of alleles amplified by these microsatellite markers ranged from 4 to 12 (average, 5.7). The mean *H*_O_ and *H*_E_ were 0.510 and 0.720, respectively.

The genetic diversity of *C. pumila* has been shown to be similar to or higher than that of the halophyte *Spergularia media* (*H*_O_: 0.546, *H*_E_: 0.698) [48] and other species such as *Carex kobomugi* (*H*_O_: 0.648, *H*_E_: 0.451) [23], *Carex macrocephala* (*H*_O_: 0.073, *H*_E_: 0.523) [24], and *Carex scabrifolia* (*H*_O_: 0.350, *H*_E_: 0.419) [49] of the genus *Carex*.

The average PIC value of the markers was 0.660 (range 0.474–0.864). The mean PIC values of *C. pumila* ranged from 0.5 to 0.8, indicating moderate to high polymorphism. Analyzing the genetic diversity of populations and developing plant cultivars requires microsatellite polymorphisms, and microsatellite markers have been successfully used for this purpose [50,51]. The markers developed in this study show a high PIC value of 0.5 or higher, which will be useful for evolutionary and population genetic analysis of populations.

## 4. Conclusions

This study is the first to report the characterization of *C. pumila* genomic information. Assembly of its genome generated a draft genome assembly of *C. pumila* based on PacBio Sequel II and Illumina paired-end sequencing. The draft genome was assembled from 2941 contigs with an estimated genome size of 0.346 Gb. The estimate of repeats in the genome was 31.0%, and heterozygosity ranged from 0.426 to 0.441%. The integrity evaluation of the assembly revealed 1481 complete BUSCOs (91.76%) in Embryophyta odb10, indicating the high quality of the draft assembly. A total of 23,402 protein-coding genes were successfully predicted and annotated in the protein database. UpsetR plots showed that 7481 orthogroups were shared by all species. The phylogenetic tree showed that *C. pumila* is a close but remote relative of *A. comosus*. Among the 25 plant genomes, there were significant gene family expansions (2–4005) and contractions (5–4267) (*p* < 0.01). *C. pumila* had a greater contraction (3154) than expansion (392). Among the extended gene families, aquaporins have been found to be enriched. The average number of alleles amplified by the 30 polymorphic markers was 4–12, with an average PIC value of 0.660, which we believe will be useful for evolutionary and genetics analyses. We conclude that these markers can strongly support genetic diversity analysis and cultivar development studies as basic data. In conclusion, our study provides a useful resource for comparative genomics, phylogeny, and future population studies of *C. pumila*.

## Figures and Tables

**Table 1:** Summary information of raw data for long- and short-read sequencing in *C. pumila*.

Short Reads	Total Reads	Total Reads Length (bp)	Q20 (%)	Q30 (%)
Raw data	870,146,384	131,392,103,984	92.31	83.52
Filtered data	350,433,302	52,800,516,376	99.47	97.17
			N50 (bp)	Mean subread length (bp)
Subreads data	33,136,805	127,339,898,260	5273	3842
Subreads (Iso-seq)	18,257,235	24,044,828,716	1462	1317

**Table 2:** Summary information of genome assembly of *C. pumila*.

Assembler	HGAP	Wtdbg2
Contig	2941	4385
Total contig bases (bp)	346,579,715	338,223,786
N50 (bp)	351,713	160,342
Max. length (bp)	1,780,279	1,030,000
Average length (bp)	117,844	77,131
Min. length (bp)	1345	1661
GC contents (%)	33.4	33.9

**Table 3:** Benchmarking universal single-copy orthologs analysis results of the two assembly methods.

Status	HGAP	Wtdbg2
Complete BUSCO (C)	1481 (91.76%)	1390 (86.12%)
Complete and single-copy BUSCO (S)	1416 (87.73%)	1367 (84.70%)
Complete and duplicated BUSCO (D)	65 (4.02%)	23 (1.43%)
Fragmented BUSCO (F)	31 (1.92%)	76 (4.71%)
Missing BUSCO (M)	102 (6.32%)	148 (9.17%)
Total BUSCO groups searched	1614 (100%)	1614 (100%)

**Table 4:** Summary of protein gene predictions and annotations for *C. pumila*.

	Numbers of Genes	Length (bp) and Match Gene (%)
Predicted proteins	23,402	35,450,136 bp
EggNOG	22,464	95.99
InterPro	18,762	80.17
Pfam	19,190	82.00
COG	9741	41.62
KEGG	8667	37.04

**Table 5:** Microsatellite information screened from the *C. pumila* genome sequence.

Result of Microsatellites Search	
Total number of contigs sequences examined	2941
Total size of examined sequences (bp)	346,579,715
Total number of identified microsatellites	62,565
Number of microsatellites containing contigs sequences	2328
Di-nucleotides	55,751
Tri-nucleotides	22,650
Tetra-nucleotides	6970
Penta-nucleotides	3628
Hexa-nucleotides	3162

**Table 6:** Characteristic and diversity information for the 30 microsatellite loci of *C. pumila*.

Locus	GenBank Accession No.	Primer Sequences (5′−3′)	Repeat Motif	Annealing Tm (°C)	Fluorescent Labels	Product Size	*N*	*N*_A_	*H*_O_	*H*_E_	PIC
CapuMS10	OQ685862	F: TGTAAAACGACGGCCAGTTGTTTATGGAAGCTTCTCCTGGT R: CGGTTCCGATCGACGTTTTG	AT(42)	57	FAM	373	24	6	0.292	0.688	0.617
CapuMS12	OQ685863	F: TGTAAAACGACGGCCAGTGGATGGACACCCCTCAACAA R: CCAACAAAAGACGCTCAGCA	AT(40)	57	VIC	278	24	4	0.750	0.646	0.586
CapuMS14	OQ685864	F: TGTAAAACGACGGCCAGTTCAATGGTCTGGAAACAACGA R: CGGTTTGGATCGACAGTTTGG	TA(39)	57	FAM	374	24	4	0.375	0.680	0.604
CapuMS21	OQ685865	F: TGTAAAACGACGGCCAGTTCCAATGAAACCAGGTGCCT R: GCACAGGACCTCAGGAACAA	TA(38)	57	VIC	286	24	8	0.250	0.741	0.691
CapuMS23	OQ685866	F: TGTAAAACGACGGCCAGTCGCACTGCACGACTTTTGAA R: AACGGTTCGGATCGACTGTT	AT(36)	57	VIC	267	24	4	0.958	0.666	0.585
CapuMS27	OQ685867	F: TGTAAAACGACGGCCAGTTGGAGACATGATGGAGGCAT R: CAAGCCACTGGGGATTCTCA	TA(32)	57	FAM	352	24	8	0.125	0.780	0.728
CapuMS43	OQ685868	F: TGTAAAACGACGGCCAGTGCCGTAGCACGATAACAACC R: CCATAGCACCGTAGCCAAGT	AT(27)	57	NED	324	24	4	0.250	0.622	0.560
CapuMS45	OQ685869	F: TGTAAAACGACGGCCAGTTAGCACCCCAGCCTAACTCT R: TGTGATTTGGTTGCACCGTT	AT(26)	57	VIC	265	24	5	0.375	0.712	0.654
CapuMS48	OQ685870	F: TGTAAAACGACGGCCAGTTCCAGTTGCGAGTCACTTCC R: GTCGTAGCACCGTAGCTCTC	AT(24)	57	VIC	210	24	6	0.542	0.829	0.785
CapuMS56	OQ685871	F: TGTAAAACGACGGCCAGTTTGGACGGTGTACCTGCTTC R: TTCATTGGTTCGGTCCCTCT	TTC(30)	57	VIC	211	24	12	0.875	0.895	0.864
CapuMS57	OQ685872	F: TGTAAAACGACGGCCAGTTGTCTTCTTCATGCATGTTCGC R: AGAAATGGGGCTGTCTTCGT	AAT(30)	57	FAM	374	24	6	0.458	0.559	0.516
CapuMS59	OQ685873	F: TGTAAAACGACGGCCAGTGACGGCTATCGATCGAGCTT R: AGACAGTGACGTGATCCTCA	TTA(30)	57	PET	153	24	9	0.458	0.847	0.808
CapuMS61	OQ685874	F: TGTAAAACGACGGCCAGTCGGTCTCGCTCTACTTGCTT R: GCCATGTACCGGTGTCTGAT	TTA(28)	57	NED	339	24	6	0.375	0.802	0.752
CapuMS62	OQ685875	F: TGTAAAACGACGGCCAGTTGAGAGGTCAAAACGGAGTGA R: ATCCGATCCGCTACTGTGTC	TAT(27)	57	PET	196	24	8	0.792	0.839	0.796
CapuMS64	OQ685876	F: TGTAAAACGACGGCCAGTAGCAAGCTAGCCCTTTGACT R: AACGATTGGTACTCTGAGCA	TAT(26)	57	VIC	214	24	4	0.375	0.630	0.554
CapuMS66	OQ685877	F: TGTAAAACGACGGCCAGTCCAGAAGTGGGGTTTGATTTGG R: TGTCCAAGTGTCAGTTTCCAAC	ATA(26)	57	VIC	316	24	8	0.792	0.856	0.818
CapuMS68	OQ685878	F: TGTAAAACGACGGCCAGTAGCCGTAGTACCGTAGTCCT R: AAGGCTTTTCACCCTGTCAA	ATA(25)	57	VIC	323	24	6	0.792	0.723	0.670
CapuMS71	OQ685879	F: TGTAAAACGACGGCCAGTCACTTTCGGTTGGTGCGATT R: ATAAGCGCAGACCTGTGACA	TTA(24)	57	NED	312	24	5	0.333	0.670	0.605
CapuMS73	OQ685880	F: TGTAAAACGACGGCCAGTTCGGAGTCCCTCTCTTCCTT R: ACACCATTGTCATACTAGCCAGA	TAT(22)	57	NED	304	24	7	0.667	0.696	0.644
CapuMS76	OQ685881	F: TGTAAAACGACGGCCAGTAGCCAGCAAAACTTAATCACGA R: TTTCAATCTCGGCCGTTGGA	ATA(21)	57	PET	178	24	5	0.833	0.752	0.691
CapuMS77	OQ685882	F: TGTAAAACGACGGCCAGTAAACAGTCGTGTCACCATCT R: GGACAGATCCGGACACCATT	ATA(20)	57	NED	328	24	5	0.125	0.623	0.544
CapuMS84	OQ685883	F: TGTAAAACGACGGCCAGTCGGAGGACAAGATGAACGGT R: CGGGAATAACACCGTCTGCTA	TCTA(10)	57	FAM	363	24	4	0.375	0.719	0.647
CapuMS85	OQ685884	F: TGTAAAACGACGGCCAGTTCTGAGCTGTACGCATTCCC R: TTGTAGCACCGTAGCCCTTT	ATAC(10)	57	FAM	353	24	5	0.750	0.719	0.653
CapuMS88	OQ685885	F: TGTAAAACGACGGCCAGTCCGAGTCATGTGCACCACTA R: TCTGAACGGGGCAAGTATGT	TTAT(9)	57	NED	303	24	5	0.333	0.773	0.716
CapuMS89	OQ685886	F: TGTAAAACGACGGCCAGTTGCCACCTGTACGTGTAGTG R: GCACCGCTGGACACTACATA	AAAT(9)	57	NED	332	24	4	0.792	0.554	0.474
CapuMS90	OQ685887	F: TGTAAAACGACGGCCAGTCACCACGGTTCCGAAACAAA R: AGCAACGTCTACCATTGGCA	ACAT(9)	57	PET	180	24	4	0.583	0.699	0.633
CapuMS95	OQ685888	F: TGTAAAACGACGGCCAGTCACCCTCCAATCCATCACGA R: GACCGACATCGAGTGAAGGA	CTGGG(10)	57	FAM	371	24	6	0.250	0.730	0.669
CapuMS96	OQ685889	F: TGTAAAACGACGGCCAGTCTGGGCTGATGTCAGGTTGT R: CCACACGCGTGCATTTAATCT	GTATT(8)	57	VIC	291	24	5	0.708	0.708	0.640
CapuMS97	OQ685890	F: TGTAAAACGACGGCCAGTGGTCACTCATGTCTCCGTCA R: CGGAGGCAAAGCTTGAACAA	ACACC(8)	57	VIC	237	24	4	0.583	0.720	0.651
CapuMS99	OQ685891	F: TGTAAAACGACGGCCAGTTGTGGGTGCATCAGAGACAC R: TGGGACGTCTAGGGGACAAT	CATGC(8)	57	VIC	287	24	4	0.125	0.708	0.637

*N*: number of samples, *N*_A_: number of alleles, *H*_O_: observed, *H*_E_: expected heterozygosity, PIC: polymorphism information content.

## Data Availability

The data presented in this study are available in the Materials and Methods section. Publicly available datasets were generated in this study. These data can be found at the following locations: the assembled genomic sequence has been deposited in the GenBank WGS database, accession number, JARJHM000000000; the developed microsatellite loci have been deposited in GenBank OQ685862-OQ685891.

## Supplementary Materials

The following supporting information can be downloaded at: https://www.mdpi.com/article/10.3390/genes14112063/s1, Figure S1: Microsatellite information of *C. pumila* using MISA tool analysis; Table S1: Twenty-five species of information used in OrthoFinder for orthologous groups analysis; Table S2: OrthoFinder statistics of 25 species with *C. pumila*; Table S3: Gene ontology (GO) terms enrichment analysis of the expanded gene families of orthologous groups in *C. pumila*.
