# Supplementary material for: Genome Assembly and Microsatellite Marker Development Using Illumina and PacBio Sequencing in the Carex pumila (Cyperaceae) from Korea
Source: Genes (Basel). 2023 Nov 10;14(11):2063. doi: 10.3390/genes14112063 (PMC10671310; doi:10.3390/genes14112063)
Supplement: Supplementary file 1 [file genes-14-02063-s001.zip › genes-2606050-supplementary.pdf]

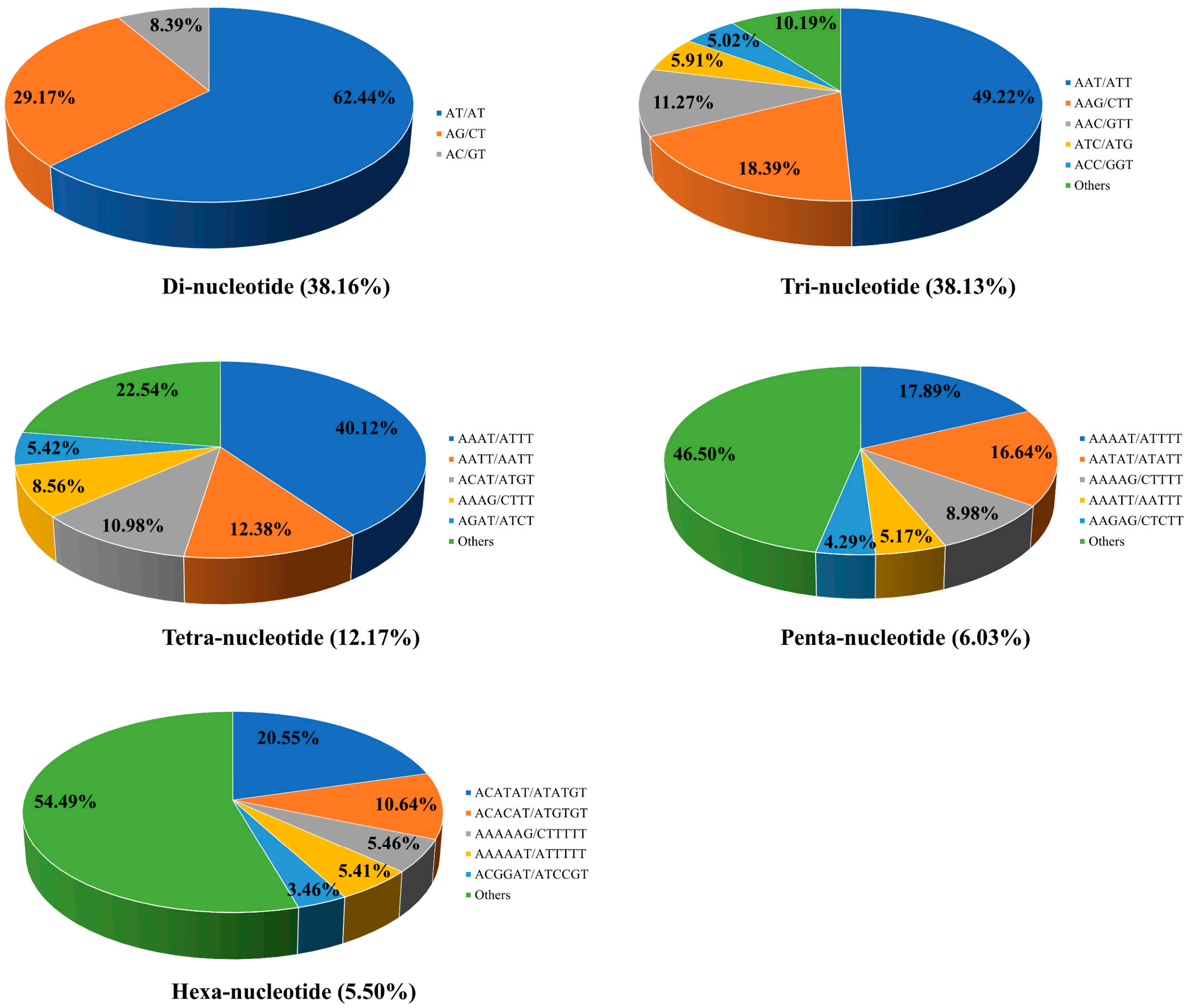

Figure S1. Microsatellite information of *C. pumila* using MISA tool analysis.

Table S1. Twenty-five species of information used in OrthoFinder for orthologous groups analysis.

Scientific name	GenBank WGS accession numbers
Digitaria exilis	GCA015342445.1
Zizania palustris	GCA019279435.1
Eleusine coracana	GCA021604985.1
Triticum turgidum	GCA900231445.1
Miscanthus lutarioriparius	GCA904845875.1
Arabidopsis thaliana	GCF000001735.4
Sorghum bicolor	GCF000003195.3
Brachypodium distachyon	GCF000005505.3
Oryza glaberrima	GCF000147395.1
Oryza brachyantha	GCF000231095.2
Setaria italica	GCF000263155.2
Oryza sativa	GCF001433935.1
Ananas comosus	GCF001540865.1
Triticum dicoccoides	GCF002162155.2
Panicum hallii	GCF002211085.1
Aegilops tauschii	GCF002575655.2
Triticum urartu	GCF003073215.2
Setaria viridis	GCF005286985.1
Panicum virgatum	GCF016808335.1
Triticum aestivum	GCF018294505.1
Lolium perenne	GCF019359855.1
Lolium rigidum	GCF022539505.1
Zea mays	GCF902167145.1
Carex pumila	This study
Hordeum vulgare	GCF904849725.1

Table S2. OrthoFinder statistics of 25 species with *C. pumila*.

Statistics	
Number of species	25
Number of genes	1,469,587
Number of genes in orthogroups	1,418,513
Number of unassigned genes	51,074
Percentage of genes in orthogroups	96.5
Percentage of unassigned genes	3.5
Number of orthogroups	60,498
Number of species-specific orthogroups	23,000
Number of genes in species-specific orthogroups	90,498
Percentage of genes in species-specific orthogroups	6.2
Mean orthogroup size	23.4
Median orthogroup size	5.0
G50 (assigned genes)	66

G50 (all genes)	63
O50 (assigned genes)	6,184
O50 (all genes)	6,580
Number of orthogroups with all species present	7,481

Table S3. Gene ontology (GO) terms enrichment analysis of the expanded gene families of orthologous groups in *C. pumila*.

GO Terms	Type	Function	Percentage of Genes
GO:0005623	Cellular component	cell	10.8
GO:0044464	Cellular component	cell part	10.8
GO:0005622	Cellular component	intracellular	9.8
GO:0044424	Cellular component	intracellular part	9.8
GO:0012505	Cellular component	endomembrane system	2
GO:0042175	Cellular component	nuclear outer membrane-endoplasmic reticulum membrane network	1
GO:0031975	Cellular component	envelope	1
GO:0071944	Cellular component	cell periphery	1
GO:0030312	Cellular component	external encapsulating structure	1
GO:0044422	Cellular component	organelle part	3.9
GO:0044446	Cellular component	intracellular organelle part	3.9
GO:0031090	Cellular component	organelle membrane	2
GO:0043226	Cellular component	organelle	6.9

GO:0043229	Cellular component	intracellular organelle	6.9
GO:0044422	Cellular component	organelle part	3.9
GO:0043227	Cellular component	membrane-bounded organelle	5.9
GO:0043228	Cellular component	non-membrane-bounded organelle	1
GO:0032991	Cellular component	protein-containing complex	5.9
GO:0099023	Cellular component	tethering complex	1
GO:0098796	Cellular component	membrane protein complex	2
GO:1990904	Cellular component	ribonucleoprotein complex	1
GO:0120114	Cellular component	Sm-like protein family complex	1
GO:1902494	Cellular component	catalytic complex	1
GO:0005839	Cellular component	proteasome core complex	1
GO:0033588	Cellular component	Elongator holoenzyme complex	1
GO:0005576	Cellular component	extracellular region	3.9
GO:0048046	Cellular component	apoplast	2
GO:0016020	Cellular component	membrane	11.8
GO:0044425	Cellular component	membrane part	5.9
GO:0031090	Cellular component	organelle membrane	2
GO:0005789	Cellular component	endoplasmic reticulum membrane	1
GO:0042175	Cellular component	nuclear outer membrane-endoplasmic reticulum membrane network	1
GO:0044425	Cellular component	membrane part	5.9
GO:0098796	Cellular component	membrane protein complex	2
GO:0005789	Cellular component	endoplasmic reticulum membrane	1
GO:0031224	Cellular component	intrinsic component of membrane	3.9
GO:0005488	Molecular function	binding	34.3

GO:1901363	Molecular function	heterocyclic compound binding	21.6
GO:0097159	Molecular function	organic cyclic compound binding	21.6
GO:0043167	Molecular function	ion binding	22.5
GO:0048037	Molecular function	cofactor binding	3.9
GO:0036094	Molecular function	small molecule binding	11.8
GO:0005515	Molecular function	protein binding	8.8
GO:0044877	Molecular function	protein-containing complex binding	1
GO:0097367	Molecular function	carbohydrate derivative binding	10.8
GO:0008144	Molecular function	drug binding	8.8
GO:0060089	Molecular function	molecular transducer activity	1
GO:0038023	Molecular function	signaling receptor activity	1
GO:0003824	Molecular function	catalytic activity	37.3
GO:0016491	Molecular function	oxidoreductase activity	7.8
GO:0016787	Molecular function	hydrolase activity	8.8
GO:0016740	Molecular function	transferase activity	16.7
GO:0140096	Molecular function	catalytic activity, acting on a protein	8.8
GO:0016829	Molecular function	lyase activity	1
GO:0098772	Molecular function	molecular function regulator	2
GO:0030234	Molecular function	enzyme regulator activity	2
GO:0005215	Molecular function	transporter activity	5.9
GO:0022857	Molecular function	transmembrane transporter activity	5.9
GO:0016209	Molecular function	antioxidant activity	1
GO:0004601	Molecular function	peroxidase activity	1
GO:0051179	Biological process	localization	8.8

GO:0051234	Biological process	establishment of localization	8.8
GO:0051641	Biological process	cellular localization	1
GO:0033036	Biological process	macromolecule localization	1
GO:0032502	Biological process	developmental process	1
GO:0048856	Biological process	anatomical structure development	1
GO:0032501	Biological process	multicellular organismal process	2
GO:0007275	Biological process	multicellular organism development	1
GO:0044706	Biological process	multi-multicellular organism process	1
GO:0008152	Biological process	metabolic process	32.4
GO:0006807	Biological process	nitrogen compound metabolic process	17.6
GO:0071704	Biological process	organic substance metabolic process	24.5
GO:0009058	Biological process	biosynthetic process	9.8
GO:0044237	Biological process	cellular metabolic process	23.5
GO:0042440	Biological process	pigment metabolic process	1
GO:0044238	Biological process	primary metabolic process	21.6
GO:0044281	Biological process	small molecule metabolic process	4.9
GO:0009056	Biological process	catabolic process	2.9
GO:0019222	Biological process	regulation of metabolic process	2
GO:0019748	Biological process	secondary metabolic process	1
GO:0055114	Biological process	oxidation-reduction process	7.8
GO:0009987	Biological process	cellular process	26.5
GO:0044237	Biological process	cellular metabolic process	23.5
GO:0007154	Biological process	cell communication	2
GO:0008037	Biological process	cell recognition	1

GO:0071554	Biological process	cell wall organization or biogenesis	2.9
GO:0051716	Biological process	cellular response to stimulus	1
GO:0050794	Biological process	regulation of cellular process	2.9
GO:0007165	Biological process	signal transduction	1
GO:0016043	Biological process	cellular component organization	2.9
GO:0050896	Biological process	response to stimulus	2.9
GO:0006950	Biological process	response to stress	1
GO:0042221	Biological process	response to chemical	1
GO:0051716	Biological process	cellular response to stimulus	1
GO:0051704	Biological process	multi-organism process	1
GO:0044706	Biological process	multi-multicellular organism process	1
GO:0022414	Biological process	reproductive process	1
GO:0009856	Biological process	pollination	1
GO:0009875	Biological process	pollen-pistil interaction	1
GO:0048544	Biological process	recognition of pollen	1
GO:0000003	Biological process	reproduction	1
GO:0022414	Biological process	reproductive process	1
GO:0050789	Biological process	regulation of biological process	2.9
GO:0050794	Biological process	regulation of cellular process	2.9
GO:0019222	Biological process	regulation of metabolic process	2
GO:0023052	Biological process	signaling	1
GO:0007165	Biological process	signal transduction	1
GO:0065007	Biological process	biological regulation	2.9
GO:0050789	Biological process	regulation of biological process	2.9

GO:0071840	Biological process	cellular component organization or biogenesis	3.9
GO:0016043	Biological process	cellular component organization	2.9
GO:0044085	Biological process	cellular component biogenesis	2
